# Inaugural Editorial

**DOI:** 10.1089/tmr.2020.28999.edi

**Published:** 2020-11-18

**Authors:** Elizabeth A. Krupinski

It is with great enthusiasm that I write this editorial for the inaugural edition of *Telemedicine Reports*. I have been involved in telemedicine for over 30 years and must admit that we are currently in what I would consider the most exciting of those 30 years. Not only is telemedicine at the forefront of healthcare as a critical and necessary way for patients and providers to stay connected in times of the public health emergency, but it is finally also actively on the agenda of world governments, healthcare payers, administrators, educators and patients. We have arrived!

Why do we need another journal? There are a number of highly respected journals dedicated to telehealth, telemedicine, m-Health, e-Health and a host of related topics, and nearly every clinical specialty journal either has a dedicated telemedicine or related section these days or at least regularly publishes articles on the topic.

The goal of *Telemedicine Reports* is to be the leading open-access, peer-reviewed journal for telemedicine research, education, and implementation science reports. The emphasis of the journal will be on published research on the successful development and integration of telehealth technologies and applications into healthcare settings, with the goal of improving the efficacy, efficiency, and effectiveness of improving patient health outcomes. We are particularly interested in multidisciplinary and global research and education efforts, reflecting the diversity of patient populations, healthcare systems, and research methodologies impacted by telehealth. *Telemedicine Reports* will bring healthcare to anyone, anywhere, anytime as a fully open access publication. Our goal is to disseminate research results online and free of cost to readers in the most timely fashion.

As an open access, online journal, *Telemedicine Reports* is not bound to the traditional model of collecting articles into issues and only releasing them periodically. Authors can submit papers for full peer-review, receive comments in a timely fashion for quick revision turn-around if the paper is deemed suitable for publication, and expect to see the final article online and available for the public to read in far less time than with a more traditional journal. There is so much going on in telehealth and telemedicine today that waiting months for cutting-edge research and information to appear in print is simply not acceptable. *Telemedicine Reports* will be the go-to journal for state-of-the-art information on a host of important and highly relevant topics that will help propel telemedicine to even further heights.

I am looking forward to heading this amazing effort and new publication from the Mary Ann Liebert Inc. publishers. I will soon be welcoming a group of world experts to the *Telemedicine Reports* Editorial Board to help with this undertaking and to guide the journal in its progress and efforts. For all of you interested in and conducting research in telehealth, comparative effectiveness, human factors and implementation science, healthcare educators, trainers & curriculum developers, medical informatics, and telehealth technology, I welcome you to *Telemedicine Reports* and encourage you to submit to, read and refer to our new journal!

